# The evolution of dual antiplatelet therapy in patients with coronary artery disease

**DOI:** 10.1093/ehjopen/oeag107

**Published:** 2026-07-04

**Authors:** Moa Simonsson, Stefan James, Jurriën M ten Berg, Bruna Gigante

**Affiliations:** Department of Medicine Solna, Division of Cardiology, Karolinska Institutet, D1:04, SE-171 76, Stockholm, Sweden; Department of Medical Sciences, Uppsala University, Uppsala University Hospital, SE-751 85 Uppsala, Sweden; Cardiovascular Research Institute Maastricht, Maastricht University Medical Center, P.O Box 616, 6200 MD Maastricht, the Netherlands; Department of Cardiology, St.Antonius Hospital, Koekoekslaan 1, 343 CM Nieuwegein, the Netherlands; Department of Medicine Solna, Division of Cardiology, Karolinska Institutet, D1:04, SE-171 76, Stockholm, Sweden

**Keywords:** Dual Antiplatelet Therapy, Acute Coronary Syndrome, Chronic coronary syndrome, antiplatelet strategy

## Abstract

Dual antiplatelet therapy (DAPT) with aspirin and a P2Y12 inhibitor has been the mainstay of therapy for acute coronary syndrome (ACS) and chronic coronary syndrome (CCS) following percutaneous coronary intervention (PCI) for more than two decades. Over this period, numerous trials have evaluated different antiplatelet strategies, initially focusing on more effective and prolonged regimens, and more recently on decreasing the intensity or duration of DAPT. Despite a wealth of accumulated evidence, the optimal antiplatelet strategy—including the ideal agent, combination, or treatment duration—remains a subject of active debate. This review summarizes the evolution of antiplatelet therapy, with particular emphasis on randomized controlled trials of DAPT in patients with ACS or CCS undergoing PCI.

## Graphical Abstract

**Graphical Abstract:**
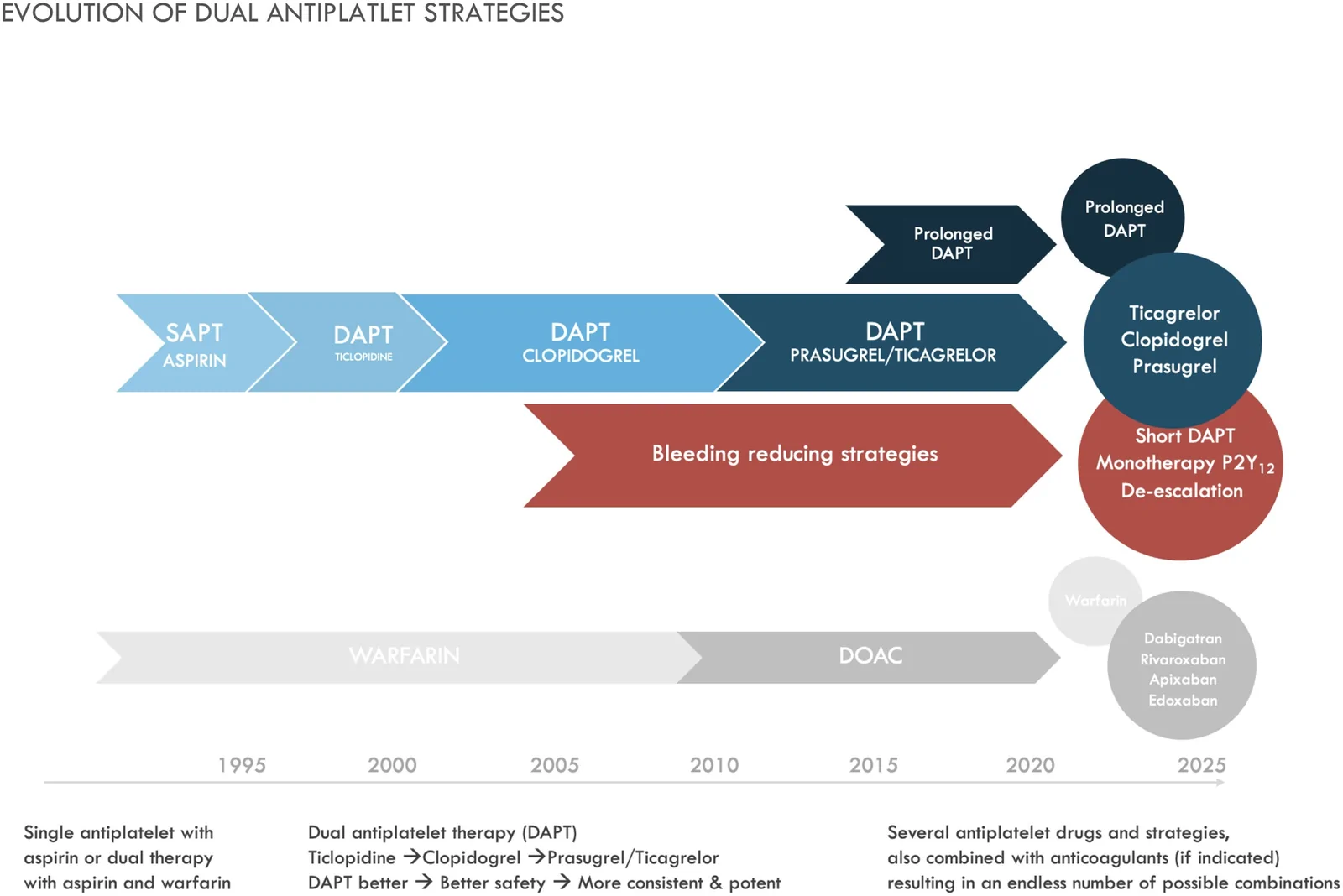
Randomized trials of dual antiplatelet therapy strategies from 1995 to 2025. The size of the bubbles represents the number of patients in each study. The colour indicates the antiplatelet agent or strategy studied. The position on the x-axis represents the year of publication, while the position on the y-axis was chosen solely for visual spacing.

## Introduction

Acute coronary syndrome (ACS) most commonly arises from rupture or erosion of an atherosclerotic plaque, triggering platelet activation, thrombus formation, and coronary artery occlusion with downstream myocardial ischaemia.^1^ Percutaneous coronary intervention (PCI) with stent implantation restores coronary blood flow in ACS and improves myocardial perfusion in chronic coronary syndrome (CCS). While stents maintain vessel patency and improve outcomes, they also promote platelet activation and thrombosis. Consequently, antiplatelet therapy has become fundamental to the management of ACS and the prevention of recurrent events, including stent thrombosis after PCI, forming the basis for dual antiplatelet therapy (DAPT). This review describes the evolution of antiplatelet therapy from the discovery of aspirin to contemporary treatment strategies, focusing on DAPT in patients with ACS or CCS following PCI (*Central Illustration*). Antiplatelet therapy in patients undergoing coronary artery bypass grafting (CABG) is beyond the scope of this review.

## Aspirin

Acetylsalicylic acid, synthesized in 1897 and marketed as aspirin, has long been used for its analgesic and antipyretic properties. Aspirin irreversibly inhibits cyclooxygenase-1 (COX-1), thereby reducing thromboxane A2 synthesis and platelet aggregation.^2^ Clinical trials in the late 20th century,^3–5^ including the ISIS-2 trial,^6^ established aspirin as the cornerstone therapy for acute myocardial infarction (MI). Notably, these early trials evaluated only short treatment durations of 4–6 weeks.

## The emergence of PCI and the challenge of stent thrombosis

In 1977, Andreas Gruntzig performed the first PCI using balloon angioplasty for a significant left anterior descending artery stenosis in a patient with stable angina.^7^ The subsequent introduction of coronary stents reduced restenosis but introduced the challenge of stent thrombosis. Early studies of bare-metal stents reported a high incidence of stent thrombosis, most often within 2 weeks.^8^ In these early studies, antithrombotic regimens were not prespecified; patients typically received heparin followed by a vitamin K antagonist (VKA) and antiplatelet therapy (aspirin, dipyridamole, or sulfinpyrazone). These findings highlighted the limited protection provided by early antithrombotic strategies against stent thrombosis.

## Ticlopidine

The first-generation thienopyridine ticlopidine irreversibly inhibits the platelet P2Y12 receptor (adenosine diphosphate receptor). It is a prodrug requiring hepatic metabolism for conversion to its active form.^9^

In the mid-1990s, ticlopidine-based DAPT for 4–6 weeks was shown to be superior to both aspirin plus VKA^10–14^ and aspirin alone^14^ in patients undergoing PCI for ACS^10–13^ or CCS.^10–14^ These trials demonstrated the superiority of DAPT following PCI but evaluated only short-term durations (*Table 1*). Ticlopidine use was limited by serious adverse effects, including bone marrow suppression (neutropenia, aplastic anaemia, or thrombotic thrombocytopenic purpura), as well as gastrointestinal disturbances and skin rash.

**Table 1:** Ticlopidine

Trial name Year Location	Population	Age	Blinding	Strategy	Ischaemic outcome	Bleeding outcome	Met primary outcome	Comment
**ISAR** **1996** **Germany**	*n* = 517 Successful PCI ACS 23% UAP ∼45%	62	Open-label	Ticlopidine + A vs. VKA + A	Cardiac death, MI, CABG, or repeated angioplasty at 30 days 1.6% vs. 6.2% ST 0.8% vs. 5.4%	Requiring surgery or blood transfusion, or organ dysfunction at 30 days 0% vs. 6.5%	YES superiority for ischaemic outcome (cardiac death, MI, CABG, or repeated angioplasty)	Palmaz-Schatz stent
**HALL** **1996** **Location not reported**	*n* = 226 Successful PCI Emergency 11%	58	Open-label	Ticlopidine + A 5 days followed by ticlopidine alone vs. aspirin alone	CV death, MI CABG, or repeat revascularization at 30 days 0.8% vs. 3.9% ST 0.8% vs. 2.9%	Not reported	Unclear primary endpoint No significant differences in clinical events	Six types of stents Ultrasound-guided PCI Few events
**STARS** **1998** **USA**	*n* = 1653 Successful PCI (no ACS?)	61	Open-label	Ticlopidine + A vs. VKA + A vs. aspirin alone	Death, TLR, Angio evident thrombosis or MI at 30 days 0.5% vs. 2.7% vs. 3.6%	Procedure-related requiring blood TX at 30 days 5.5% vs. 6.2% vs. 1.8%	Yes Superiority for ischaemic outcome (death, TLR, angio evident thrombosis or MI)	Bleeding was also significantly reduced
**FANTASTIC** **1998** **Europe**	*n* = 236 PCI unplanned 42%	60	Open-label	Ticlopidine^a^ + A^b^ vs. VKA^a^ +A^b^	Cardiac event rate at 6 weeks 5.7% vs. 8.3% (NS)	Any bleeding including haematomas at 6 weeks 13.5% vs. 21%%	Yes Superiority for bleeding	Wiktor stent
**MATTIS** **1998** **Europe**	*n* = 350 PCI high risk post AMI 18.8% UAP 39.5%	60	Open-label	Ticlopidine + A vs. VKA + A	CV death, MI, or repeat revascularization at 30 days 5.6% vs. 11% (NS)	Vascular or bleeding 1.7% vs. 6.9%	No (Trend in favour) Superiority for ischaemic outcome (CV death, MI, or repeat revascularization)	Stratified by stent type Vascular and bleeding outcomes significantly reduced

A, aspirin; ACS, acute coronary syndrome; AMI, acute myocardial infarction; BARC, Bleeding Academic Research Consortium; CABG, coronary artery bypass grafting; CV, cardiovascular; DAPT, dual antiplatelet therapy; MACE, major adverse cardiovascular events; MI, myocardial infarction; NACE, net adverse clinical outcomes; NS, non-significant; PCI, percutaneous coronary intervention; STEMI, ST-elevation myocardial infarction; ST, stent thrombosis; TIMI, thrombolysis in myocardial infarction; TLR, target lesion revascularization; UAP, unstable angina pectoris; VKA, vitamin-K-antagonist.

^a^6 weeks; ^b^life-long

## Clopidogrel

The second-generation thienopyridine clopidogrel has a similar mechanism of action as ticlopidine but a more favourable safety profile.^15^

In the landmark CURE trial^16^ (2001, *n* = 12 562), DAPT with aspirin and clopidogrel was superior to aspirin alone for the primary outcome of cardiovascular (CV) death, MI, or stroke at 1 year. The study included patients with non-ST-elevation MI (NSTEMI) or unstable angina, of whom 36% underwent PCI. DAPT duration ranged from 3 to 12 months (median 9 months).

Clopidogrel-based DAPT also demonstrated superiority to aspirin alone in patients with ST-elevation myocardial infarction (STEMI) treated with fibrinolysis^17^ and in patients with suspected ACS.^18^ Although a higher loading dose (600 mg followed by 150 mg daily) did not reduce major adverse cardiovascular event (MACE), it was associated with a lower incidence of stent thrombosis^19^ (*Table 2*). Current guidelines^20,21^ recommend either loading dose, with the lower dose of 300 mg preferred in patients receiving fibrinolysis. Although prasugrel (in patients undergoing PCI) or ticagrelor are recommended over clopidogrel in ACS, clopidogrel may be considered in elderly patients or in those at high bleeding risk.^22^ Clopidogrel is also recommended in combination with fibrinolysis or oral anticoagulants (OACs).^20,21^

**Table 2:** Clopidogrel

Trial name Year Location	Population	Age	Blinding	Strategy	Ischaemic outcome	Bleeding outcome	Met primary outcome	Comment
**CLASSICS** **2000** **Europe**	*n* = 1020 Successful PCI	60	Double-blind	Clopidogrel + A with C loading 300 mg vs. Clopidogrel + A without C loading vs. Ticlopidine + A	CV death. MI or TVR at 28 days 1.2% vs. 1.5% vs. 0.9%	Major bleeding or peripheral complication^a^ 1.5% vs. 1.2% vs. 1.2% ^a^only bleeding not reported	Yes Superiority for safety outcome (Major peripheral or bleeding complications, neutropenia, thrombocytopenia, or early discontinuation)	Safety was the primary outcome MACE was the secondary outcome
**CURE** **2001** **Global (not Asia)**	*n* = 12 562 NSTE-ACS NSTEMI 25% UAP 75% PCI 36% CABG 16.5%	64	Double-blind	Clopidogrel + A with C loading 300 mg vs. aspirin alone	CV death, MI, or stroke 9.3% vs. 11.4%	Life-threatening or major bleeding^a^ 3.7% vs. 2.7%	Yes Superiority for the ischaemic outcome (CV death MI or stroke)	DAPT duration 3 to 12 (median 9) months Majority conservatively treated (PCI 36% and CABG 16.5)
**CREDO** **2002** **USA and Canada**	*n* = 2116 PCI NSTE-ACS 67% (UAP 55%)	62	Double blinded	Clopidogrel loading 300 mg → 75 mg + A for 12 months vs. Clopidogrel loading placebo → 75 mg + A for 1 month, followed by placebo + A for 2–12 m	Death, MI, or stroke at one year 8.5% vs. 11.5% Death MI urgent revascularization at 28 days 6.8% vs. 8.3% (NS)	TIMI major bleeding at one year 8.8% vs. 6.7% (NS)	Yes Superiority for ischaemic outcome (Death MI, or stroke) Ischaemic outcome at 28 d (NS)	For the ischaemic outcome at 28 days, loading dose interaction—if before 6 h stronger trend to benefit
**CLARITY** **TIMI-28** **2005** **Global (not Asia)**	*n* = 3491 STEMI scheduled for fibrinolysis Angio within 48 to 192 h	57	Double blinded	Clopidogrel + A vs. Placebo + A (C loading 300 mg)	CV death, MI, or recurrent ischaemia leading to revascularization at 30 days 11.6% vs. 14.1%	TIMI major bleeding at 1 day after angiography or at 8 days if angiography not performed 1.3% vs. 1.1% (NS)	Yes Superiority for primary reperfusion outcome (Occluded IRA (TIMI 0 or 1), recurrent MI or death before angiography) 15% vs. 21%	Primary outcome ‘failed reperfusion or re-occlusion’ Median 84 h to angiography Short follow-up primary endpoint at 3.5 days
**COMMIT** **2005** **China**	*n* = 45 852 Suspected MI STEMI 87% or LBBB 6.5%	61	Double-blind	Clopidogrel + A vs. Placebo + A	Death, MI, or stroke in-hospital or up 28 days (mean 15 days 9.2% vs. 10.1% Death alone 7.5% vs. 8,1%	Fatal, cerebral, or transfusion (at 28 days?) 0.58% vs. 0.55%	Yes Superiority for ischaemic outcome (Death, MI, or stroke in-hospital or up 28 days)	No loading doses of C or A Fibrinolysis 54% (no PCI?) Elective PCI 3%
**CURRENT-OASIS-7** **2010** **Global**	*n* = 25 086 ACS intended PCI STEMI 29%	61.4	Double-blind for C Open-label for A	Clopidogrel 600 mg + 150 mg day 2–7 → 75 mg vs. Clopidogrel 300 mg → 75 mg A_high_ 300–325 mg vs. A _low_ 75–100mg	CV death, MI, or stroke at 30 days 4.2% vs. 4.4% A_high_ vs. A_low_ 4.2% vs. 4.4%	Major bleeding 2.5% vs. 2.0% TIMI major 1.7% vs. 1.3% A_high_ vs. A_low_ 2.3% vs. 2.3%	No Superiority for ischaemic outcome (CV death, MI, or ST and bleeding outcome were significant	ST significantly reduced in the PCI cohort (1.6% vs. 2.3%), and bleeding was significantly increased No difference in the aspirin comparison

A, aspirin; ACS, acute coronary syndrome; BARC, Bleeding Academic Research Consortium; CV, cardiovascular; CVD, cardiovascular disease; DAPT, dual antiplatelet therapy; LBBB, left bundle branch block; MACE, major adverse cardiovascular events; MI, myocardial infarction; NACE, net adverse clinical outcomes; NSTE-ACS, non-ST elevation acute coronary syndrome; PCI, percutaneous coronary intervention; RF, risk factor; STEMI, ST-elevation myocardial infarction; ST, stent thrombosis; TIMI, thrombolysis in myocardial infarction; TVR, target vessel revascularization; UAP, unstable angina pectoris.

^a^Cure bleeding definition (life-threatening; fatal, Hb drop >5 g/dL, requiring surgery, intracranial, requiring inotropic agents, or major bleeding; requiring transfusion of ≥2 U of blood)

## Limitations of clopidogrel

Clopidogrel is a prodrug that requires hepatic activation through a two-step process, mainly dependent on CYP2C19. Genetic polymorphisms in CYP2C19 influence the response to clopidogrel, resulting in measurable interindividual variability. Approximately 20% of patients are heterozygous carriers of loss-of-function alleles, resulting in reduced formation of the active metabolite, while about 3% are homozygous carriers with minimal or absent active metabolite formation.^23,24^ Consequently, the antiplatelet effect of clopidogrel is variable, and the onset of action is slower (2–6 h)^25^ than that of prasugrel or ticagrelor.

## Prasugrel

The third-generation thienopyridine prasugrel has a faster onset of action and provides more potent and consistent platelet inhibition than clopidogrel. Prasugrel is a prodrug that is metabolized in both the intestine and the liver and does not depend on CYP2C19 for activation.^26^ (*Table 3*)

**Table 3:** Prasugrel and ticagrelor

Trial name Year Location	Population	Age	Blinding	Strategy	Ischaemic outcome	Bleeding outcome	Met primary outcome	Comment
**Prasugrel**								
**Triton TIMI 2007** **Global (not Asia)**	*n* = 13 608 ACS planned for PCI STEMI 26%	61	Double-blind	Prasugrel + A vs. Clopidogrel + A	CV death, MI, or stroke at 14.5 months 9.9% vs. 12.1%	TIMI major bleeding (non-CABG) 2.4% vs. 1.8% life-threatening 1.4% vs. 0.9% Fatal 0.4% vs. 0.1%	Yes Superiority for ischaemic outcome (CV death, MI, or stroke)	Post hoc analyses: Previous stroke/TIA—Net harm Elderly 75 years or underweight <60 kg—No Benefit
**Triology ACS 2012** **Global**	7 243 NSTE-ACS aged <75 and managed medically	62	Double-blind	Prasugrel + A vs. Clopidogrel + A Loading doses C-20 mg and A-300 mg if <72 h from ACS diagnosis Prasugrel 5 mg if weight <60 kg or age ≫75 years	CV death, MI, or stroke at 30 months 13.9% vs. 16.0% NS	GUSTO Severe or life-threatening non-CABG bleeding 0.4% vs. 0.4% TIMI major non-CABG at 30 months 2.1% vs. 1.5%	No Superiority for ischaemic outcome (CV death, MI, or stroke)	Full study population 9326. Primary outcome among patients <75 years (*n* = 7243), exploratory analysis for patients ≫75 years
**ACCOAST** **2013** **Global (not Asia)**	*n* = 4033 NSTEMI scheduled for angiography within 2–48h PCI performed 68.7% CABG 6.2%	64	Double-blind	Prasugrel loading 30 mg + 30 mg at PCI vs. Placebo + 60 mg at PCI	CV death, MI, stroke, urgent revascularization, GPIIb/IIIa bailout at 7 days 10% vs. 9.8%	All TIMI major 2.6% vs. 1.4% Non-CABG-related TIMI major 1.3% vs. 0.5%	No Superiority for ischaemic outcome (CV death, MI, stroke, urgent revascularization or GP bailout)	Median time from loading to PCI in pretreatment group 4.3 h Femoral access 57%
**Ticagrelor**								
**Plato** **2009** **Global**	n = 18 624 ACS STEMI 38% Angio 81.5% PCI 61%	62	Double-blind	Ticagrelor + A vs. C + A	CV death, MI, or stroke at 12 months 9.8% vs. 11.7%	PLATO non-CABG related at 12 months 4.5% vs. 3.8% TIMI major non-CABG related 2.8% vs. 2.2%	Yes Superiority for ischaemic outcome (CV death, MI, or stroke)	Interaction for region, signal of no benefit in North America, suggested related to higher aspirin dosing
**Atlantic** **2014** **Global (not Asia)**	1 862 STEMI	61	Double-blind	Prehospital vs. hospital ticagrelor loading	Absence of ≫70% ST-resolution before PCI or absence of TIMI 3 flow in IRA	PLATO major 1.8% vs. 1.6% TIMI major 1.3% vs. 1.3%	No Superiority for ischaemic outcome (Absence of ≫70% ST-resolution before PCI or absence of TIMI 3 flow in IRA)	Treatment difference 31 min. Secondary outcome of ST at 30 days 0.2% vs. 1.2% GPIIb/IIIa 28.7%
**Prasugrel versus ticagrelor**								
**Prague-18** **2018** **Czech Republic** **1-year outcome publication**	*n* = 1230 Primary or immediate PCI STEMI 90% LBBB 5% NSTEMI 5%	62	Open-label	Prasugrel + A vs. Ticagrelor + A	CV death, MI, or stroke at 1 year 6.6% vs. 5.7%	TIMI major 0.9% vs. 0.7% BARC major 2.4% vs. 1.5%	No Superiority for ischaemic outcome (CV death, MI, or stroke)	Terminated early for futility. 34% and 44% switched to clopidogrel after a median of 8 days
**ISAR-REACT 5** **2019** **Germany and** **Italy**	*n* = 4018 ACS STEMI 41% NSTEMI 46% PCI 84%	65	Open-label	Prasugrel-based vs. Ticagrelor-based strategy	Death, MI, or stroke at 12 months 6.9% vs. 9.3%	BARC 3,4 or 5 bleeding 4.8% vs. 5.4% (NS)	Yes Superiority for ischaemic outcome (death, MI, or stroke)	Both ischaemic and bleeding outcomes in favour of prasugrel
**TUXEDO-2** **2025/2026** **India**	*n* = 1800 DM + multivessel disease + PCI with stent ACS 79%	60	Open-label	Prasugrel + A vs. Ticagrelor + A	Death, MI, or stroke 8.6% vs. 10.3% (NS)	Major bleeding 7.1% vs. 8.4%	No Non-inferiority for NACE (Death, MI, stroke, or BARC major bleeding) 14.2% vs. 16.6%	Lower protocol-adherence to prasugrel than ticagrelor (98.8% vs. 91.6% at discharge and 85% vs. 81.6% at 12 months)

A, aspirin; ACS, acute coronary syndrome; BARC, Bleeding Academic Research Consortium; CV, cardiovascular; DAPT, dual antiplatelet therapy; DM, diabetes mellitus; IRA, infract related artery; MI, myocardial infarction; LBBB, left bundle branch block; NACE, net adverse clinical outcomes; NS, non-significant; NSTE-ACS, non-ST elevation acute coronary syndrome; NSTEMI, non-ST-elevation myocardial infarction; PCI, percutaneous coronary intervention; STEMI, ST-elevation myocardial infarction; ST, stent thrombosis; TIMI, thrombolysis in myocardial infarction.

In the landmark TRITON-Thrombolysis in Myocardial Infarction (TIMI) 38 trial^27^ (2007, *n* = 13 608), patients with ACS scheduled for PCI were randomized to prasugrel- or clopidogrel-based DAPT. In non-ST-elevation acute coronary syndrome (NSTE-ACS), coronary anatomy had to be known and suitable for PCI before prasugrel initiation, whereas in STEMI, or NSTE-ACS with known coronary anatomy, prasugrel could be administered before PCI but no more than 24 h prior to the procedure.

Prasugrel-based DAPT reduced the primary outcome of CV death, MI, or stroke compared with clopidogrel, but increased bleeding, including life-threatening and fatal bleeding events. A *post hoc* analysis demonstrated net harm in patients with prior stroke or transient ischaemic attack (TIA) and no net benefit in patients ≥75 years or those with body weight (<60 kg).

In the TRILOGY trial^28^ (2012, *n* = 7243, age <75years), prasugrel-based DAPT in patients with ACS managed medically without PCI showed no benefit compared with clopidogrel-based DAPT. Prasugrel pretreatment in patients with NSTE-ACS scheduled for coronary angiography was subsequently evaluated in the ACCOAST trial^29^ (2013, *n* = 4033). Patients were randomized to a prasugrel 30 mg loading dose before angiography, followed by an additional 30 mg at PCI, or placebo followed by a full 60 mg prasugrel loading dose at PCI. Prasugrel pretreatment did not reduce ischaemic outcomes but significantly increased major bleeding. The short median pretreatment interval of 4.3 h may not reflect the time between NSTEMI diagnosis and invasive management in clinical practice.

## Ticagrelor

Ticagrelor is a cyclopentyltriazolopyrimidine that acts through reversible, non-competitive binding to the P2Y12 receptor. Unlike thienopyridines, ticagrelor is directly active, with faster onset and more potent and consistent antiplatelet effects than clopidogrel. It also has an active metabolite, AR-C124910XX^30^ (*Table 3*).

The landmark PLATO trial^31^ (2009, *n* = 18 624) included invasively or medically managed ACS patients. Ticagrelor-based DAPT was superior to clopidogrel-based DAPT for the primary outcome of CV death, MI, or stroke at 12 months as well as all-cause death. While trial-defined major bleeding (including CABG-related bleeding) was similar, non-CABG-related bleeding was significantly increased.

In the ATLANTIC trial^32^ (2014, *n* = 1862), prehospital vs. in-hospital ticagrelor loading in STEMI did not reduce the co-primary endpoints of absence of ≥70% ST-segment elevation resolution before PCI or absence of TIMI 3 flow at angiography but reduced the secondary endpoint of stent thrombosis at 30 days. No difference in major bleeding was observed. The modest difference in loading time of 31 min may have attenuated the treatment effect, as the onset of ticagrelor is delayed in STEMI.

## Clinical indications and labelling

These differences in efficacy and safety are reflected in the regulatory labelling for prasugrel and ticagrelor. Use of prasugrel is restricted to patients with ACS undergoing PCI and, in NSTE-ACS, to those with known coronary anatomy. It is contraindicated in patients with a history of stroke or TIA, and should be used with caution, with dose reduction in elderly patients (≥75 years) and those with low body weight (<60 kg).

Ticagrelor, in contrast, is indicated for all ACS patients irrespective of invasive or conservative strategy. In NSTE-ACS, ticagrelor may be initiated at the time of suspected diagnosis, independent of the timing of coronary angiography.

## Prasugrel versus ticagrelor

Although prasugrel and ticagrelor are distinct compounds, their *in vitro* antiplatelet effects are similar, and both have a class I recommendation in ACS guidelines.^20,21^ However, comparisons between the two drugs, or between treatment strategies based on their respective indications, have been of considerable interest. Due to differences in labelling, direct comparisons are possible in STEMI, whereas in NSTE-ACS, only indirect comparisons between the two treatment strategies are possible (*Table 3*),

Prasugrel- vs. ticagrelor-based DAPT was evaluated in the PRAGUE-18 trial^33^ (2016, *n* = 1230), which enrolled patients undergoing primary PCI for STEMI (90%), newly diagnosed left bundle branch block (5%), or immediate PCI for NSTEMI (5%). The study was terminated early for futility after enrolling fewer than half of the planned 2500 patients. There was no difference in the primary outcome of all-cause death, MI, stroke, bleeding requiring transfusion or prolonging hospitalization, or urgent target vessel revascularization within 7 days, nor in the secondary ischaemic or bleeding outcomes. At 1 year, no significant differences in ischaemic or bleeding outcomes were observed. However, due to a lack of reimbursement for study drugs, a substantial proportion of patients switched to clopidogrel^34^ (34% in the prasugrel and 44% in the ticagrelor group).

A prasugrel- vs. ticagrelor-based strategy was subsequently evaluated in the ISAR-REACT 5 trial^35^ (2019, *n* = 4018), which enrolled patients with ACS scheduled for PCI. Patients with STEMI were randomized to open-label prasugrel or ticagrelor, while patients with NSTE-ACS were randomized to an open-label prasugrel- or ticagrelor-based strategy, with ticagrelor loading at randomization and prasugrel loading in the catheterization laboratory after the decision to proceed to PCI. The difference in loading time was 55 min, and 98.7% vs. 86.1% received the planned loading dose in the ticagrelor and prasugrel groups, respectively. The primary outcome of all-cause death, MI, or stroke at 1 year occurred more frequently with ticagrelor than prasugrel. There was no significant difference in major bleeding, although bleeding rates were numerically higher with ticagrelor.

A similar strategy was evaluated in patients with type 1 or type 2 diabetes mellitus and multivessel coronary artery disease undergoing PCI as a tertiary objective in the TUXEDO-2 trial^36^ (2026, *n* = 1800). Ticagrelor failed to meet non-inferiority for the primary outcome of all-cause death, MI, stroke, or Bleeding Academic Research Consortium (BARC) major bleeding at 1 year. Although differences were not statistically significant, ticagrelor was associated with numerically higher rates of both bleeding and ischaemic outcomes.

The 2023 European Society of Cardiology (ESC) ACS guidelines introduced a class IIa recommendation favouring prasugrel over ticagrelor in patients with ACS undergoing PCI, primarily based on the ISAR-REACT 5 trial, a recommendation not adopted in the 2025 ACC/AHA ACS guidelines.

## Origins and rationale for the 12-month DAPT recommendation in ACS

None of the landmark studies, CURE, TRITON, or PLATO, support a 12-month duration of DAPT, as this was not specifically studied in these trials. The median DAPT duration ranged from 9 months in CURE and PLATO to 15 months in TRITON. Importantly, none of these trials evaluated DAPT duration; CURE compared DAPT vs. no DAPT, whereas TRITON and PLATO compared clopidogrel-based DAPT with prasugrel- or ticagrelor-based DAPT, respectively.

In August 2006, at the ESC congress, two studies reporting an increased risk of late stent thrombosis with first-generation drug-eluting stents (DES) sparked intense debate. In response, the FDA convened an advisory panel in December 2006, which issued a consensus statement recommending at least 12 months of DAPT following PCI with DES.^37^ Since then, 12 months of DAPT for patients with ACS has been the default strategy in both ESC^20^ and ACC/AHA guidelines.^21^ However, although many subsequent studies have used 12 months of DAPT as the standard treatment, this strategy has often not been superior to alternative approaches and does not provide robust support for a fixed 12-month DAPT regimen.^38^ A recent expert consensus document on antithrombotic treatment for secondary prevention in ACS reinforced the importance of individualized strategies over a fixed-duration approach.^39^

## Prolonged DAPT

Acknowledging the residual risk of ischaemic events beyond 1 year after ACS^40^ or PCI,^41,42^ prolonged DAPT has been evaluated in two large randomized controlled trials (*Table 4*). The DAPT study^43^ (2014, *n* = 9961), supported by the FDA, enrolled patients 12 months after PCI and compared continuation of DAPT with a thienopyridine, predominantly clopidogrel, vs. aspirin alone from 12 to 30 months. Continued treatment with a thienopyridine reduced the primary outcome of death, MI, stroke, or stent thrombosis at 18 months (30 months after the index event) but increased the risk of moderate and severe bleeding. The reduction in ischaemic events was primarily driven by lower rates of stent thrombosis and MI, with no difference in CV death. An increase in non-CV death was observed, which in a *post hoc* analysis was attributed to a baseline imbalance in patients with known cancer.

**Table 4:** Prolonged DAPT

Trial name Year Location	Population	Age	Blinding	Strategy	Ischaemic outcome	Bleeding outcome	Met primary outcome	Comment
**DAPT STUDY** **Global (not Asia)**	*n* = 9961 PCI with stent, event-free and adherent at 12m Indication for PCI AMI 26% STEMI 10%	62	Double-blind	Continued thienopyridine + A vs. Placebo + A (clopidogrel, 65%, prasugrel 35%)	Death, MI, or stroke at from 12 to 30 months 4.3% vs. 5.9% ST at 30 months 0.4% vs. 1.4%	BARC 2,3 or 5 bleeding 5.9% vs. 2.9% GUSTO severe or moderate 2.5% vs. 1.6%	YES Superiority for ischaemic outcome (death, MI, or stroke)	Of 25 682 enrolled at index PCl, 44% were event-free, adherent, and randomized at 12 months Increased non-CV death Suggested due to baseline imbalance in patients with cancer
**PEGASUS TIMI-54** **2015** **Global**	*n* = 21 162 Previous MI within 1 to 3 (median 1.7) years STEMI 53%	65	Double-blind	Ticagrelor 90 mg × 2 + A vs. Ticagrelor 60 mg × 2 + A vs. Placebo + A	CV death, MI, or stroke at 3 years 7.9% vs. 7.8% vs. 9.0%	TIMI major 2.6% vs. 2.3% vs. 1.1%	YES Superiority for the ischaemic outcome (CV death, MI, or stroke)	Similar absolute increase in bleeding as absolute decrease in ischaemic events

A, aspirin; AMI, acute myocardial infarction; BARC, Bleeding Academic Research Consortium; CV, cardiovascular; DAPT, dual antiplatelet therapy; MI, myocardial infarction; PCI, percutaneous coronary intervention; STEMI, ST-elevation myocardial infarction; ST, stent thrombosis; TIMI, thrombolysis in myocardial infarction.

DAPT with ticagrelor 90 mg or 60 mg twice daily was evaluated in patients with prior MI within 3 years (median 1.7 years) in the PEGASUS trial^44^ (2015, *n* = 21 162). DAPT with either dose of ticagrelor reduced the primary outcome of CV death, stroke, and MI at 3 years but increased the risk of bleeding. Efficacy was similar between ticagrelor doses, but the 60 mg dose was better tolerated and associated with numerically lower bleeding rates.

## Bleeding as a prognostic factor and advances in stent techniques

One of the first reports linking major bleeding to increased mortality came from the GRACE registry (2003).^45^ This association has since been replicated in multiple cohorts,^46–50^ showing a strong dose-dependent relationship between bleeding events and mortality. Consistent with these findings, the OASIS-5,^51^ HORIZONS,^52^ and MATRIX^53^ trials demonstrated that reductions in bleeding were associated with lower mortality. In parallel, advances in PCI techniques, including thinner and more advanced stents and the use of intracoronary imaging,^54^ have reduced the risk of stent-related complications, enabling less intensive antiplatelet therapy. Bleeding is now recognized as a key prognostic outcome, and several antithrombotic strategies aimed at reducing bleeding without increasing ischaemic events have been evaluated.

## The first aspirin-free strategies in combination with OAC

**Figure 1:**
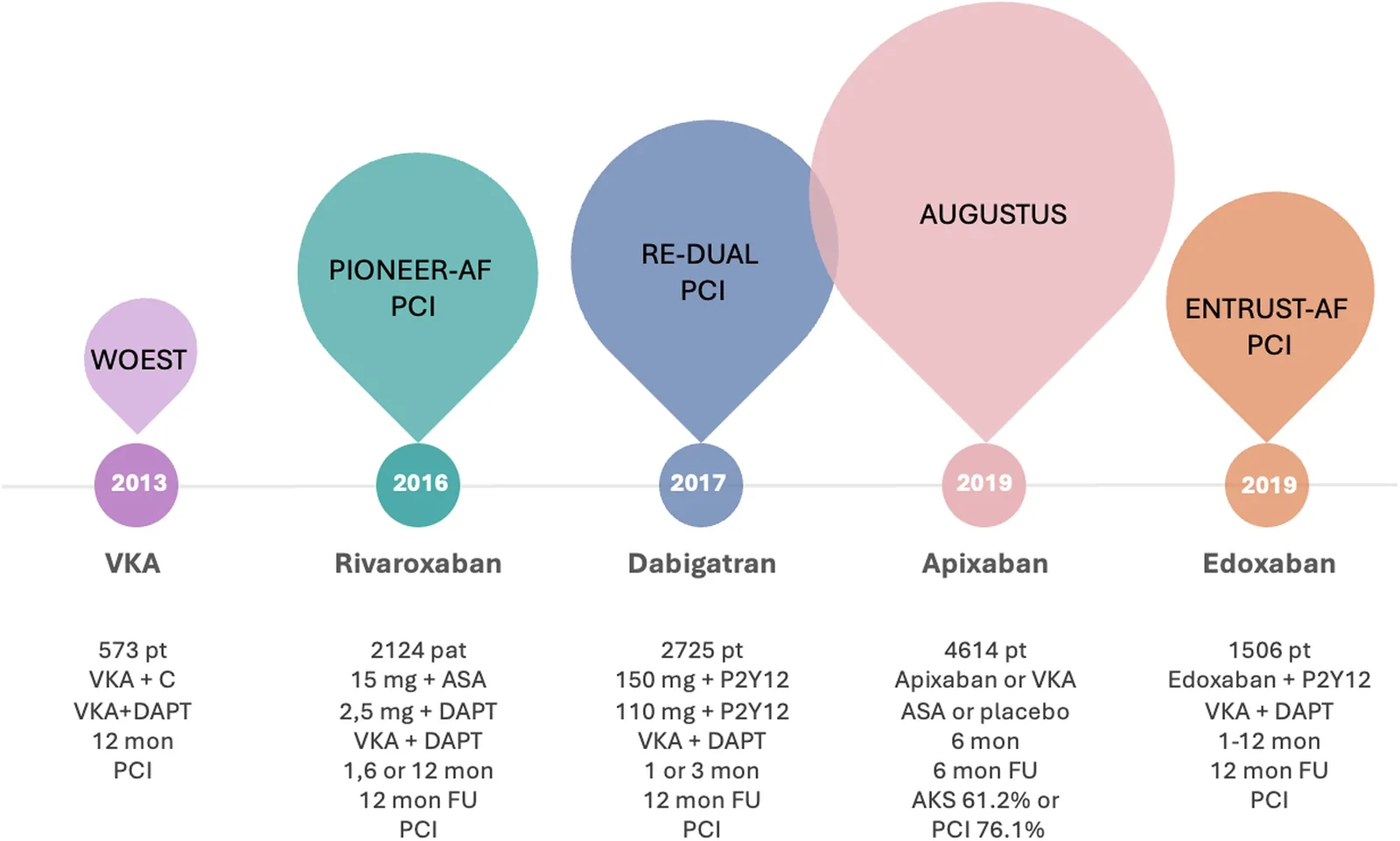
Trials of combination therapy with antiplatelets and oral anticoagulants. The size of the bubbles represents the number of patients in each study. The colour of the balloons indicates the antiplatelet agent or strategy studied, and the position on the *x*-axis represents the year of publication.

Since the 1980s, aspirin has formed the basis of antithrombotic therapy in coronary heart disease. Although it has only been evaluated for short durations after MI, its use has been routinely extended without clear supporting evidence until recently. Discontinuation of aspirin rather than the P2Y12 inhibitor was first introduced in patients undergoing PCI for ACS or CCS who had a concomitant indication for OAC therapy, irrespective of indication, in the WOEST trial^55^ (2013, *n* = 573). Dual therapy with VKA and clopidogrel was superior to triple therapy with VKA and DAPT (clopidogrel + aspirin) for the primary outcome of any bleeding at 12 months. No signal of harm was observed, but there was a reduction in the secondary composite ischaemic outcome (*Table 5* and *Figure 1*).

**Table 5:** Combination therapy

Trial name Year Location	Population	Age year	Blinding	Strategy	Ischaemic outcome	Bleeding outcome	Met primary outcome	Comment
**WOEST** **2013** **Netherlands** **Belgium**	*n* = 573 PCI + OAC ACS 27.5% OAC indication: AF 69% MHV 10% Other 20%	70	Open-label	Dual: Clopidogrel + VKA vs. Triple: Clopidogrel + VKA + A	Death, MI stroke, urgent revascularization, or ST 11.1% vs. 17.6%	Any bleeding at 1 year 19.4% vs. 44.4% TIMI major 3.2% vs. 5.6% (NS)	Yes Superiority for Any bleeding	Randomization before and up to 4 h after PCI No power for major bleeding or ischaemic events
**PIONEER AF-PCI** **2013** **Global**	*n* = 2124 PCI + AF STEMI 12.3%	70	Open-label	Dual: Rivaroxaban 15 mg + Clopidogrel (1 or 6) months vs. Triple experimental: Rivaroxaban 2.5 mg × 2 + Clopidogrel + A vs. Triple standard: VKA + Clopidogrel + A	CV death, MI, or stroke at 12 months 6.5% vs. 5.6% vs. 6.0%	TIMI major, minor, or requiring medical attention at 12 months 16.8% vs. 18.0% vs. 26.7% TIMI major 2.1% vs. 1.9% vs. 3.3% (NS)	Yes Superiority for bleeding (TIMI major, minor, or requiring medical attention)	Randomization within 72 h Broad bleeding definition as primary outcome Off-label doses of rivaroxaban Different DAPT durations (1.6 or 12 m)
**REDUAL-PCI 2017** **Global**	*n* = 2725 PCI + AF ACS 51%	69 110 mg 72 150 mg	Open-label	Dual: Dabigatran 110 mg × 2 + P2Y_12_ vs. Dual: Dabigatran 150 mg × 2 + P2Y_12_ vs. Triple: VKA + P2Y_12_ + A	MI, stroke, systemic embolism, death, or unplanned revascularization 15.2% vs. 13.4% (110 mg) 11.8% vs. 12.8% (150 mg)	ISTH major or CRNM bleeding at 12 months 15.4% vs. 26.9% (110 mg) 20.2% vs. 25.7% (150 mg) TIMI Major Bleeding 1.4% vs. 3.8% (110 mg) 2.1% vs. 3.9% (150 mg)	Yes Superiority for bleeding (ISTH major or CRNM bleeding) Yes Non-inferiority dual vs. triple for thromboembolic endpoints and unplanned revascularization	Randomization within 5 days Ticagrelor 12% Major bleeding significantly reduced All ischaemic endpoints numerically higher with 110 mg Different comparisons for 110 and 150 mg doses
**AUGUSTUS** **2019** **Global**	*n* = 4616 ACS or PCI ACS + PCI 37.3% Only ACS 23.9% Only PCI 38.8%	71	Open-label Double-blind for aspirin	R: 2 × 2 Apixaban + P2Y_12_ vs. VKA + P2Y_12_ A + P2Y_12_ vs. placebo + P2Y_12_	Death or ischaemic event 6.7% vs. 7.1% Apixaban vs. VKA 6.5% vs. 7.3% A vs. placebo Not tested statistically	ISTH major or CRNM bleeding at 6 months 10.5% vs. 14.7% Apixaban vs. VKA 16.1% vs. 9.0% A vs. placebo TIMI major bleeding 1.7% vs. 2.1% Apixaban vs. VKA 2.4% vs. 1.3% A vs. placebo	Yes Non-inferior, or superior for bleeding (ISTH major or CRNM) Met both non-inferiority superiority	Randomization within 14 days median 6 days Clopidogrel 92.6% Follow up 6 months Numeric increase in MI and ST in the no aspirin arm
**ENTRUST-AF PCI 2019** **Global**	*n* = 1506 PCI + AF ACS 52%	70	Open-label	Dual: Edoxaban + P2Y_12_ vs. Triple: VKA + P2Y_12_ + A	CV death, MI, ST, stroke, or systemic embolism 7% vs. 6%	ISTH major or CRNM bleeding at 12 months 17% vs. 20% ISTH Major bleeding 6% vs. 6%	Yes and no Hierarchical non-inferiority and then superiority for ISTH major or CRNM bleeding Met non-inferiority but not superiority	45 h to randomization 92% clopidogrel 7.5% ticagrelor < 1% prasugrel Non-proportional hazard with early increase in bleeding and curves crossing with edoxaban

A, aspirin; ACS, acute coronary syndrome; AF, atrial fibrillation; BARC, Bleeding Academic Research Consortium; C, clopidogrel, CRNM, clinically relevant nonmajor; DAPT, dual antiplatelet therapy; IRA, infract related artery; ISTH, Internation Society on Thrombosis and Haemostasis; MI, myocardial infarction; MHV, mechanical heart valve; NS, non-significant; OAC, oral anticoagulant; PCI, percutaneous coronary intervention; R, randomization; STEMI, ST-elevation myocardial infarction; ST, stent thrombosis; TIMI, thrombolysis in myocardial infarction; VKA, vitamin-K-antagonist.

### Direct oral anticoagulants

The trials of rivaroxaban,^56^ dabigatran,^57^ and edoxaban^58^ included patients undergoing PCI with non-valvular atrial fibrillation and no other indication for OAC (*Table 5* and *Figure 1*). In contrast, the apixaban trial^59^ (AUGUSTUS 2019, *n* = 4614) included patients with ACS (with or without PCI) or CCS undergoing PCI, of whom 24% had ACS managed medically. It was the only trial to directly compare a direct oral anticoagulant (DOAC) with warfarin using a 2 × 2 factorial design, randomizing patients to apixaban vs. VKA and aspirin vs. placebo, and it demonstrated a benefit of apixaban at 6 months.

The other three DOAC trials^56–58^ compared dual therapy with a DOAC with triple therapy with VKA with 12-month follow-up. Notably, the low-dose rivaroxaban 2.5 mg twice daily used in the PIONEER AF-PCI trial^56^ (2016, *n* = 2124) is not approved for stroke or systemic embolic prevention, whereas the rivaroxaban 15 mg dose is recommended for patients with reduced renal function, except in some Asian countries where it has broader approval.

Despite different trial designs, omission of aspirin consistently reduced bleeding across trials.^60^ While none of the trials were powered for ischaemic outcomes and no significant increase in composite ischaemic events was observed, a signal of increased ischaemic risk was noted, particularly for MI and stent thrombosis.^60^ The very short duration of triple therapy, ranging from 2 to 6 days, may have contributed to this signal. Clopidogrel was predominantly used as a P2Y12 inhibitor, whether interindividual variability in response^24^ contributed is unclear, as neither platelet function testing nor genotyping was performed.

Current guidelines uniformly recommend a very short period of triple therapy, followed by dual therapy with OAC (preferably a DOAC) and a P2Y12 inhibitor (preferably clopidogrel) for 6 to 12 months, depending on the bleeding risk, and thereafter OAC alone.^20,21,61^

## Antiplatelet strategies to reduce bleeding in patients without an OAC indication

**Figure 2:**
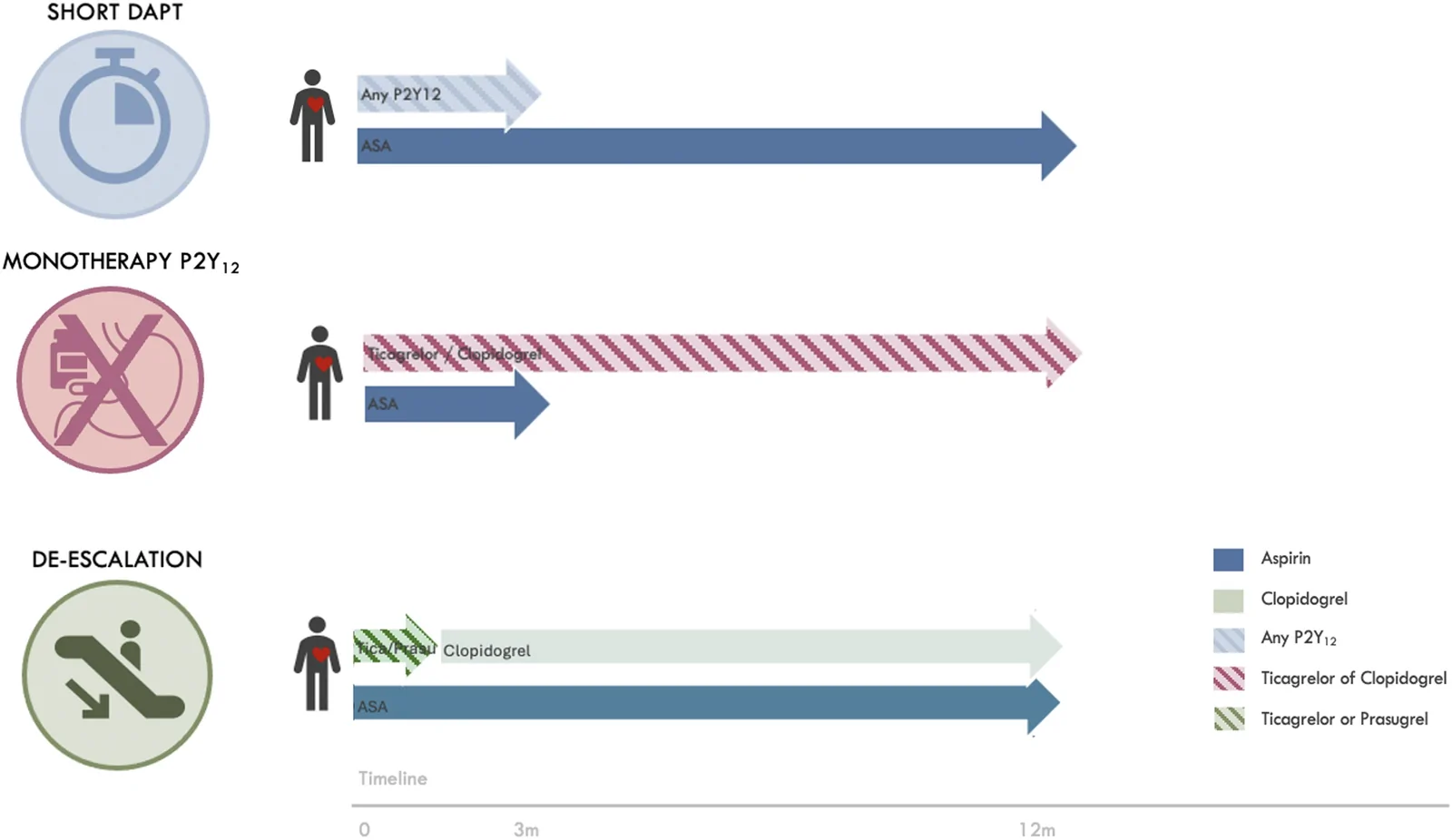
The three bleeding-reducing strategies. Short DAPT (top), monotherapy with P2Y12 inhibitor (middle), and de-escalation (bottom). The colour of the arrows represents the antiplatelet agent, and the length of the arrows represents the treatment duration.

These strategies can be divided into three categories^62^ (*Figure 2*).

1. Short DAPT: After 1, 3, or 6 months of DAPT, the P2Y12 inhibitor is discontinued, and aspirin monotherapy is continued.
2. P2Y12 monotherapy: After 1 or 3 months of DAPT, aspirin is discontinued, and P2Y12 inhibitor monotherapy is continued.
3. De-escalation: DAPT with aspirin plus ticagrelor or prasugrel is de-escalated to DAPT with clopidogrel or lower-dose prasugrel.

### Short DAPT

Short DAPT (3 or 6 months) followed by aspirin monotherapy has been evaluated in several trials^63–68^ (*Figure 2* and *Table 6*). Most were small and powered for non-inferiority using net adverse clinical events (NACE). NACE may be a problematic endpoint in antithrombotic trials, as opposing trends in ischaemic and bleeding outcomes can bias results towards non-inferiority, and non-inferiority margins are often arbitrary. In the PRODIGY trial (*n* = 1551), 6 vs. 12 months of DAPT was not superior at 24 months for the primary ischaemic outcome of death, MI, or cerebrovascular events. A meta-analysis^69^ of six trials demonstrated that longer DAPT was associated with increased bleeding. However, in patients with ACS, 3 months, compared with longer durations, was associated with increased ischaemic risk, leaving the question of optimal DAPT duration unresolved.

**Table 6:** Short versus longer DAPT with clopidogrel

Trial name Year Location	Population	Age	Blinding	Strategy	Ischaemic outcome	Bleeding outcome	Met primary outcome	Comment
**EXCELLENT** **2012** **South Korea**	*n* = 1443 PCI STEMI 3% NSTE-ACS 48%	63	Open-label	6 vs. 12 months DAPT Clopidogrel as P2Y_12_inhibitor	Cardiac death, MI, or ischaemia-driven TVR at 12 months 4.8% vs. 4.3% ST 0.9% vs. 0.1%	TIMI major bleeding 0.3% vs. 0.6%	Yes Non-inferiority (cardiac death, MI, or ischaemia-driven TVR)	Low event rate and small sample size Numerically more MI, ST, and target vessel MI with short DAPT
**RESET** **2012** **South Korea**	*n* = 2117 PCI AMI 14% UAP 40%	62	Open-label	3 months DAPT + E-ZES stent vs. 12 months DAPT + other stents Clopidogrel as P2Y_12i_nhibitor	Death, MI, or stent thrombosis at 12 months 0.8% vs. 1.3% (NS)	TIMI Major bleeding 0.2 vs. 0.6%	Yes Non-inferiority for NACE (CV death, MI, stent thrombosis or TVR or bleeding) 4.7% vs. 4.7%	Low event rate and small sample size
**PRODIGY** **2012** **Italy**	*n* = 1970 PCI ACS 74% STEMI 33%	69	Open-label	6 vs. 24 months DAPT Clopidogrel as P2Y_12_inhibitor	Death, MI, or cerebrovascular accident 10.% vs. 10.1% (NS)	TIMI major 0.6% vs. 1.6% BARC 3 or 5 1.9% vs. 3.4%	No Superiority for ischaemic outcome (Death, MI, or CV accident)	Randomization at one month
**OPTIMIZE 2013** **Brazil**	*n* = 3119 PCI + CCS or low-risk ACS NSTEMI 5%	62	Open-label	3 vs. 12 months DAPT	Death, MI, or stroke at 12 months 5.6% vs. 5.1%	Major bleeding at 12 months 0.6% vs. 0.9%	Non-inferiority for NACE (Death, MI, stroke, or major bleeding) 6.8% vs. 7.0%	Low-risk population Study specific definition of major bleeding
**SECURITY** **2014** **Spain, Italy and Netherlands**	*n* = 1399 PCI UAP 38%	65	Open-label	6 vs. 12 months DAPT Clopidogrel as P2Y_12_inhibitor	Events at 12m: Cardiac death 0.7% vs. 0.4% MI 2.3% vs. 2.1% Stroke 0.9% vs. 0.3% ST 0.3% vs. 0.4%	BARC type 3 or 5 bleeding at 12 months 0.6% vs. 1.1%	Yes Non-inferiority for NACE (cardiac death, MI, stroke, ST, or BARC 3 or 5 bleeding) 3.7% vs. 4.5%	Low risk population Few events
**ITALIC** **2015** **Europe and Middle East**	*n* = 1850 PCI and sensitive to aspirin and event free at 6 months ACS 23%	62	Open-label	6 vs. 24 months DAPT Clopidogrel as P2Y_12_inhibitor	Death, MI, or stroke at 24 months 3.0% vs. 3.4%	TIMI major bleeding at 24 months 0% vs. 0.4%	Yes Non-inferiority for NACE (death, MI, repeat emergency TVR, stroke, or TIMI major bleeding) 3.7% vs. 3.5%	Enrolled *n* = 2031 Aspirin resistance (*n* = 137) not randomized Blanking for outcome events the first 6 months (*n* = 44) not randomized Primary PCI and left main PCI excluded Terminated premature
**META-ANALYSIS 2017**	*n* = 11 473 security, ottimize, italic, ecellent, reset, and prodigy ACS or CAD undergoing PCI with DES ACS 41.5% STEMI 5.6%	63.2	Included trials were open-label	Short ≪6 months vs. long 12 months Clopidogrel as P2Y_12_inhibitor	Cardiac death, MI, or ST 2.74% vs. 2.55%	Major bleeding 0.39% vs. 2.97%	Not applicable	ACS higher risk of ischaemic events Network MA 3 vs. 6 months 3 vs. 12 months 6 vs. 12 months 3 months DAPT increased ischaemic events in ACS pat
**SMART DATE 2018** **South Korea**	*n* = −2712 PCI for ACS STEMI (37.7%)	62	Open-label	6 vs. 12 months or longer (17.7 median) Clopidogrel as P2Y_12_inhibitor in ∼80%	All-cause death, MI, or stroke 4.7% vs. 4.2% MI: 1.8% vs. 0.6%	BARC 2 to 5 2.7% vs. 3.9% (NS) Major bleeding 0.5% vs. 0.8%	Yes Non-inferiority for ischaemic outcome (All-cause death, MI, or stroke)	Landmark analysis from 6 months 5 times increased risk of MI

ACS, acute coronary syndrome; BARC, Bleeding Academic Research Consortium; CAD, coronary artery disease; CCS, chronic coronary syndrome; DAPT, dual antiplatelet therapy; MI, myocardial infarction; NACE, net adverse clinical outcomes; NSTE-ACS, non-ST elevation acute coronary syndrome; NSTEMI, non-ST-elevation myocardial infarction; PCI, percutaneous coronary intervention; STEMI, ST-elevation myocardial infarction; ST, stent thrombosis; TIMI, thrombolysis in myocardial infarction; TVR, target vessel revascularization; UAP, unstable angina.

A similar signal was observed in the SMART-DATE trial^70^ (2018 *n* = 2712), which included patients with ACS undergoing PCI. Six months of DAPT was non-inferior to 12 months or longer with respect to the primary outcome of all-cause death, MI, or stroke. However, the non-inferiority margin was wide, and there was a significant increase in MI. Bleeding was numerically, but not significantly, reduced with 6 months of DAPT.

## Mixed short DAPT and monotherapy

Two large trials have evaluated short DAPT (1 or 3 months) followed by single antiplatelet therapy with either aspirin or a P2Y12 inhibitor. The MASTER-DAPT trial^71^ (2021, *n* = 5204) included patients event-free at 1 month after PCI with a biodegradable polymer sirolimus-eluting stent for ACS or CCS and with at least one high bleeding risk criterion. One-month DAPT was non-inferior to standard DAPT for the primary NACE outcome of all-cause death, MI, stroke, or major and clinically relevant non-major (CRNM) bleeding, and for MACE (all-cause death, MI, or stroke). Major and CRNM bleeding was reduced, with no difference in major bleeding. In the abbreviated group, 70% of the patients received a P2Y12 inhibitor (predominantly clopidogrel) (*Table 7*).

**Table 7:** Mixed short DAPT with P2Y12 or aspirin monotherapy

Trial Name year location	Population	Age	Blinding	Strategy	Ischaemic outcome	Bleeding outcome	Met primary outcome	Comment
**DUAL ACS** **2026** **Scotland (75%), England, and New Zealand**	*n* = 5052 PCI 71% CABG 6%	63	Open-label	3 vs. 12 months DAPT Clopidogrel 53% Ticagrelor 43% Prasugrel 5%	CV death or MI at 15 months 8.9% vs. 9.3% (NS)	Major bleeding 3.2% vs. 4.0% (NS)	No superiority for all-cause death	Inclusion up to 3 months after MI Halted prematurely
**MASTER DAPT 2021** **Global**	*n* = 4579 Event-free 1 month after PCI + HBR STEMI 12% NSTEMI 25% OAC 36.4%	76	Open-label	SAPT vs. standard DAPT (34 vs. 193 days)	Death. MI, or stroke at 12 months 6.1% vs. 5.9%	Major and CRNM 6.5% vs. 9.4% BARC 3 or 5 2.3% vs. 2.5%	Yes Non-inferiority for NACE (Death, MI stroke, or BARC 3 or 5 bleeding) 7.5% vs. 7.7% MACE (death, MI, or stroke) Superiority for bleeding (BARC 2, 3, or 5)	Randomization at 1 month if event-free Hierarchical testing: Non-inferiority for NACE →MACE →superiority for bleeding Mix of A and P2Y_12_ in short DAPT and OAC included

A, aspirin; ACS, acute coronary syndrome; BARC, Bleeding Academic Research Consortium; C, clopidogrel; CABG, coronary artery bypass grafting; CRNM, clinically relevant nonmajor; CV, cardiovascular; DAPT, dual antiplatelet therapy; HBR, high bleeding risk; MACE, major adverse cardiovascular events; MI, myocardial infarction; NACE, net adverse clinical outcomes; NSTEMI, non-ST-elevation myocardial infarction; OAC, oral anticoagulant; PCI, percutaneous coronary intervention; SAPT, single antiplatelet therapy; STEMI, ST-elevation myocardial infarction.

The inclusion of patients treated with OAC (36.5%) complicates interpretation as optimal DAPT duration differs with concomitant OAC therapy. The duration of triple therapy (OAC + DAPT) was 1 vs. 3 months, which is longer than recommended in current guidelines, and it is unclear whether the results can be extrapolated to other stent platforms.

In the DUAL-ACS trial (ESC 2025, not yet published), which enrolled an all-comers ACS population, 3 vs. 12 months of DAPT did not reduce the primary outcome of all-cause death at 12 months. Unlike MASTER-DAPT, the primary outcome differed from the 5-point NACE, and most patients in the short DAPT group received aspirin. The secondary MACE outcome (CV death, MI, or stroke) was numerically higher, while major bleeding was numerically lower with 3 months of DAPT. The trial was terminated prematurely after enrolling approximately one-third of the planned patients and was therefore underpowered.

## Monotherapy with P2Y12 inhibitor

Several trials have evaluated discontinuation of aspirin after an initial period of DAPT. These studies differ in patient population, duration of initial DAPT, type of P2Y12 inhibitor used for monotherapy, and timing of randomization (*Figure 2*, *Table 8*, and *Table 9*).

**Table 8:** Ticagelor-based monotherapy

Trial name Year Location	Population	Age	Blinding	Strategy	Ischaemic outcome	Bleeding outcome	Met primary outcome	Comment
**GLOBAL LEADERS** **2018** **Global**	*n* = 15 968 PCI ACS 47%	65	Open-label	Ticagrelor + A 1 month followed by Ticagrelor 23 months vs. ‘standard’ DAPT 12 months (C if CCS and ticagrelor if ACS) followed by A for 12 months	Death or Q-wave MI at 24 months 3.81% vs. 4.37% (NS)	BARC 3 or 5 at 24 months 2.04% vs. 2.12%	No Superiority for ischaemic outcome (Death or Q-wave MI)	Complex design with different comparisons first and second year One-year DAPT for CCS
**TWILIGHT** **2019** **Global**	*n* = 7119 PCI + one clinical and one angiographic high-risk feature NSTEMI 30% UAP 35%	65	Double-blind	Ticagrelor 12 months vs. Ticagrelor + A 12 months	Death, MI or stroke at 12 months 3.9% vs. 3.9%	BARC 2, 3 or 5 at 12 months 4.0% vs. 7.1% BARC 3 or 5 1.0% vs. 2.0%	Yes Superiority for Bleeding (BARC 2.3 or 5)	Of 9006 patients included 7119 (79%) were event-free + adherent and randomized at 3 months STEMI excluded CCS (29%) treated with ticagrelor
**TICO** **2020** **South Korea**	*n* = 3056 PCI + ACS STEMI 36% NSTEMI 34%	61	Open-label	Ticagrelor + A 3 months followed by Ticagrelor monotherapy 9 months vs. Ticagrelor + A 12 months	Death, MI, ST, stroke, or TVR at 12 months 2.3% vs. 3.4%	TIMI major at 12 months 1.7% vs. 3.0%	Yes Superiority for NACE (Death, MI, ST, stroke, TVR, or TIMI major bleeding) 3.9% vs. 5.9%	Low event rate First 3 months no difference in treatment or outcomes
**T-PASS** **2023** **South Korea**	*n* = 2850 PCI + ACS STEMI 40%	61	Open-label	Ticagrelor + A 16 (12–25) days followed by monotherapy up to 12 months vs. Ticagrelor + A 12 months	Death, MI stroke, ST at 12 months 1.8% vs. 2.2%(NS)	BARC 3, 4, or 5 at 12 months 1.2% vs. 3.4%	Yes Non-inferiority for NACE (Death, MI stroke, ST or BARC 3, 4 or 5 bleeding) 2.8% vs. 5.2% Superiority was also met	NACE primary outcome Low event rate
**ULTIMATE-DAPT** **2024** **China, Pakistan, UK, and Italy**	*n* = 3400 PCI STEMI 28% NSTEMI 32%	63	Double-blind	Ticagrelor + placebo vs. Ticagrelor + A	Cardiac death, MI stroke, ST, or TVR at 12 months 3.6% vs. 3.7%	BARC 2, 3 or 5 at 12 months 2.1% vs. 4.6% BARC 3 or 5 0.7% vs. 1.7%	Yes Superiority for bleeding (BARC 2, 3 or 5) Non-inferiority for ischaemic outcome (cardiac death, MI stroke, ST, or TVR)	Of 3505 included in the IVUS -ACS study, 3400 were event-free on DAPT and randomized at 1 month Low event rate
**Target first** **2025** **Europe**	*n* = 1942 PCI + ACS, STEMI 50%	61	Open-label	Any P2Y_12_ monotherapy for 12 months vs. P2Y_12_ + A for 11 months	CV death, MI, or stroke at 12 months 1.6% vs. 1.6%	BARC 2, 3 or 5 at 12 months 2.6% vs. 5.6% BARC 3 or 5 0.7% vs. 0.7%	Yes Hierarchical Non-inferiority for NACE (CV death, MI, or stroke BARC 3 or 5 bleeding) 2.1% vs. 2.2% Superiority for bleeding (BARC 2, 3, or 5)	Of 2246 patients included 1942 were event-free on DAPT and enrolled at 1 month Low event rate High-risk (complex PCI) patients excluded Ticagrelor 74% Prasugrel 21%

A, aspirin; ACS, acute coronary syndrome; BARC, Bleeding Academic Research Consortium; C, clopidogrel; CCS, chronic coronary syndrome; CV, cardiovascular; DAPT, dual antiplatelet therapy; MI, myocardial infarction; NACE, net adverse clinical outcomes; NS, non-significant; NSTEMI, non-ST-elevation myocardial infarction; PCI, percutaneous coronary intervention; STEMI, ST-elevation myocardial infarction; ST, stent thrombosis; TIMI, thrombolysis in myocardial infarction; TVR, target vessel revascularization; UAP, unstable angina.

**Table 9:** Clopidogrel-based^a^ monotherapy

Trial name Year Location	Population	Age year	Blinding	Strategy	Ischaemic outcome	Bleeding outcome	Met primary outcome	Comment
**SMART CHOICE** **2019** **South Korea**	*n* = 2993 PCI + ACS/CCS ACS 58% STEMI 11%	64	Open-label	P2Y_12_ + A for 3 months followed by mono P2Y_12_ 9 months vs. P2Y_12_ + A _12_ months	All-cause death, MI, or stroke at 12 months 2.9% vs. 2.5%	BARC 2 to 5 at 12 months 2.0% vs. 3.4% BARC 3 to 5 0.8% vs. 1.0%	YES Non-inferiority for ischaemic outcome (death, MI, or stroke)	Low event rate Clopidogrel 77% Ticagrelor 23% Numeric increase in MACE
**STOP-DAPT2** **2019** **Japan**	*n* = 3009 PCI ACS 38% STEMI 19%	69	Open-label	Clopidogrel + A for 1 month followed by Clopidogrel monotherapy vs. Clopidogrel + A for 12 months	CVdeath, MI, ST, or stroke at 12 months 1.96% vs. 2.51%	TIMI major and minor at 12 months 0.41% vs. 1.54%	YES Non-inferiority for NACE (CVdeath, MI, ST, stroke, or TIMI major and minor bleeding) 2.36% vs. 3.70% Superiority was also met	Low event rate
**STOP-DAPT2ACS** **2022** **Japan**	*n* = 4136 PCI + ACS STEMI 56% NSTEMI 20%	67	Open-label	Clopidogrel + A for 1–2 months followed by Clopidogrel monotherapy vs. Clopidogrel + A for 12 months	CV death, MI, ST, or stroke at 12 months 2.8% vs. 1.9%	TIMI major and minor at 12 months 0.5% vs. 1.2% TIMI major 0.3% vs. 0.6%	NO Non-inferiority for NACE (CV death, MI, ST, stroke, or TIMI major/minor bleeding)	3008 new + 1161 ACS patients from STOP-DAPT 2 Low event rate Numerical increase in ischaemic events, especially MI and ST
**HOST-BR** **2025** **South Korea**	*n* = 4897 1598 HBR 3299 non-HBR PCI STEMI 5%/11%	76 and 64 (HBR and non-HBR)	Open-Label	HBR: 1 vs. 3 month DAPT Non-HBR 3 vs. 12 months DAPT	Cardiac death, MI, ST, or stroke at 12 months HBR: 9.8% vs. 5.9% Non-HBR 2.2% vs. 2.3%	BARC 2, 3, or 5 at 12 months HBR: 13.8% vs. 15.8% NS Non-HBR 7.2% vs. 11.7%	NO (HBR) YES (non-HBR) Non-inferiority for NACE (Death, MI, ST, stroke, or major Bleeding) or MACE (Cardiac death, MI, ST or stroke) Superiority for bleeding (BARC 2, 3 or 5)	Clopidogrel 91%/76% MACE and NACE non-inferior in non-HBR Bleeding significantly reduced in non-HBR

A, aspirin; ACS, acute coronary syndrome; BARC, Bleeding Academic Research Consortium; C, clopidogrel; CCS, chronic coronary syndrome; CV, cardiovascular; DAPT, dual antiplatelet therapy; HBR, high bleeding risk; MI, myocardial infarction; NACE, net adverse clinical outcomes; NS, non-significant; NSTEMI, non-ST-elevation myocardial infarction; PCI, percutaneous coronary intervention; STEMI, ST-elevation myocardial infarction; ST, stent thrombosis; TIMI, thrombolysis in myocardial infarction; TVR, target vessel revascularization; UAP, unstable angina.

^a^Monotherapy with predominantly clopidogrel as P2Y12 inihibitor.

### Ticagrelor-based monotherapy

The GLOBAL LEADERS trial^72^ (2018, *n* = 15 968) was the first large study to test discontinuation of aspirin while continuing the P2Y12 inhibitor in patients with ACS or CCS undergoing PCI. In the experimental arm, aspirin was stopped after 1 month of ticagrelor-based DAPT, and ticagrelor monotherapy was continued for 23 months, irrespective of ACS or CCS. In the control arm, patients received 12 months of DAPT (ticagrelor in ACS, clopidogrel in CCS), followed by 12 months of aspirin. Superiority was not demonstrated at 24 months for the primary outcome of all-cause death or Q-wave MI. The pragmatic endpoint and the differing treatment comparisons during the first and second year may have contributed to the neutral results. Although the trial did not meet its primary endpoint, it marked the beginning of aspirin-free strategies.

Subsequently, several studies evaluating ticagrelor monotherapy after 1 or 3 months of DAPT,^73–77^ consistently demonstrated a reduction in bleeding without any signal of increased ischaemic events. The large, double-blinded TWILIGHT trial^77^ (2019 *n* = 7119) enrolled patients meeting high-risk criteria undergoing PCI for NSTE-ACS (*n* = 9006); however, only those who were event-free at 3 months (*n* = 7119) were randomized to ticagrelor monotherapy or continued DAPT. Ticagrelor monotherapy reduced the primary outcome of major or CRNM bleeding, as well as major bleeding alone, at 15 months, with no signal of increased ischaemic risk.

A shorter initial DAPT duration followed by ticagrelor monotherapy was evaluated in the T-PASS trial^73^ (2023, *n* = 2850), including patients with ACS undergoing PCI. After a median of 16 days of DAPT, ticagrelor monotherapy was non-inferior and superior to standard DAPT for the primary outcome of all-cause death, MI, stroke, stent thrombosis, or major bleeding. Major bleeding was significantly reduced, while ischaemic outcomes were similar or numerically slightly reduced.

Similar results were observed in the double-blind ULTIMATE DAPT trial^74^ (2024 *n* = 3400) in patients with ACS undergoing PCI. One month of DAPT followed by ticagrelor monotherapy significantly reduced clinically relevant bleeding (BARC 2, 3, or 5) and major bleeding (BARC 3 or 5), while ischaemic outcomes were similar.

### Clopidogrel-based monotherapy

Several trials have evaluated clopidogrel monotherapy after approximately 1 or 3 months of DAPT.^78–80^ While this strategy consistently reduced bleeding across studies, an increased risk of ischaemic events was observed in the STOPDAPT-2 ACS trial^79^ (2022 *n* = 4169), which enrolled patients with ACS undergoing successful PCI. After 1–2 months of DAPT, clopidogrel monotherapy failed to demonstrate non-inferiority for the primary outcome of CV death, MI, stroke, stent thrombosis, and major or minor bleeding at 12 months. There was a numerical increase in ischaemic events, driven mainly by higher rates of MI and stent thrombosis, alongside a reduction in bleeding events.

### Meta-analysis

A meta-analysis,^81^ encompassing 27 284 patients with ACS enrolled in the GLOBAL LEADERS,^72^ SMART CHOICE,^80^ TWILIGHT,^77^ TICO,^76^ STOPDAPT2ACS,^79^ T-PASS,^73^ and ULTIMATE DAPT trials, compared short DAPT (≤3 months) followed by P2Y12 monotherapy vs. standard 12-month DAPT. There was no difference in the primary MACE outcome at 12 months, whereas NACE, any bleeding, and major bleeding at 12 months were significantly reduced with P2Y12 monotherapy. A significant interaction by type of P2Y12 inhibitor was observed, with ticagrelor, but not clopidogrel, reducing MACE, NACE, and mortality.

## De-escalation

The third strategy does not involve shortening the duration of DAPT but rather reducing P2Y12 inhibitor intensity in patients with ACS, for whom prasugrel or ticagrelor is recommended^20,21^ (*Figure 2* and *Table 10*).

**Table 10:** De-escalation

Trial name Year Location	Population	Age	Blinding	Strategy	Ischaemic outcome	Bleeding outcome	Met primary outcome	Comment
**TROPICAL-ACS** **2017** **Europe**	*n* = 2610 ACS undergoing PCI planned 12 months DAPT STEMI 56%	59	Open-label	DAPT with A + P2Y_12_: 1w Prasugrel → 1w Clopidogrel PFT guided Clopidogrel or Prasugrel for 11.5 months vs. Standard DAPT with Prasugrel 12 months	CV death, MI or stroke at 12 months 3% vs. 3%	BARC 2–5 at 12 months 5% vs. 6%	Yes Non-inferiority for NACE (CV death, MI or stroke or BARC 2–5) 7% vs. 9%	Complicated design 40% HPR on C and switched back PFT performed in control as well
**TOPIC** **2017** **France**	*n* = 646 ACS + PCI event-free at 1 month STEMI 40%	60	Open-label	Ticagrelor/prasugrel + A for 1 month → Clopidogrel + A vs. Ticagrelor/prasugrel + A	CV death, urgent revascularize -tion stroke 9.3% vs. 11.5%	BARC ≫2 4% vs. 14.9% TIMI major 0.3% vs. 1.2% (*n* = 1 vs. *n* = 4)	Yes Superiority for NACE (CV death, urgent revascularization, stroke, or ≫ BARC 2 bleeding) 13.4% vs. 26.3%	No power for major bleeding Ticagrelor 43% Prasugrel 57%
**POPular genetics** **2019** **Netherlands**	*n* = 2488 STEMI + pPCI < 48h	62	Open-label	CYP2C19 genotyping: LOF→ C + A No LOF → Ticagrelor/prasugrel + A vs. Standard DAPT with ticagrelor/prasugrel + A	Vascular death, ACS, stroke, ST, or urgent TVR at 12 months 4.6% vs..4.7%	PLATO minor and major at 12 months 9.8% vs. 12.5% TIMI major non-CABG 1.2% vs. 1.0%	Yes Non-inferiority for NACE (death, MI, stroke, or ST or PLATO major bleeding) 5.1% vs. 5.9% Superiority for bleeding (PLATO minor and major) 9.8% vs. 12.5%	Genotyping: Clopidogrel 60% Ticagrelor 38.1% Prasugrel 1.0% Control: Clopidogrel 7% Ticagrelor 91% Prasugrel 2.3%
**HOST-REDUCE-POLYTECH-ACS** **2020** **South Korea**	*n* = 2338 ACS + PCI STEMI 14%	59	Open-label	Prasugrel 10 mg + A 1 months → prasugrel 5 mg + A 11 months vs. DAPT prasugrel 10 mg + A 12 months	Cardiac death, MI, stroke, or ST at 12 months 1.4% vs. 1.8% (NS)	BARC ≫2 at 12 months 2.9% vs. 5.9% BARC ≫3 0.8% vs. 0.7%	Yes Non-inferiority for NACE (death, MI, repeat revascularization, stroke, ST, or BARC ≫2 bleeding) 7.2% vs. 10.1%	De-escalation after one month but inclusion at index event Both non-inferiority and superiority met for primary endpoint
**TALOS-AMI** **2021** **South Korea**	*n* = 2697 AMI + PCI event-free at 1 month STEMI 54%	60	Open-label	Ticagrelor + A → Clopidogrel + A vs. Ticagrelor + A	CV death, MI, or stroke at 12 months 2.1% vs. 3.1%	BARC 2, 3, or 5 at 12 months 3.0% vs. 5.6% BARC 3 or 5 1.2% vs. 2.3%	Yes Non-inferiority for NACE (CV death, MI, stroke, or BARC 2, 3, or 5 bleeding) 4.6% vs. 8.2%	Major bleeding sign. Decreased All ischaemic outcomes numerically reduced Both non-inferiority and superiority met for primary endpoint

A, aspirin; ACS, acute coronary syndrome; AMI, acute myocardial infarction; BARC, Bleeding Academic Research Consortium; C, clopidogrel; CV, cardiovascular; DAPT, dual antiplatelet therapy; HPR, high platelet reactivity; LOF, loss-of-function; MI, myocardial infarction; NACE, net adverse clinical outcomes; NS, non-significant; PCI, percutaneous coronary intervention; pPCI, primary PCI; STEMI, ST-elevation myocardial infarction; ST, stent thrombosis; TIMI, thrombolysis in myocardial infarction.

De-escalation from prasugrel or ticagrelor to clopidogrel can be guided (based on platelet function testing or genotyping) or unguided. Guided de-escalation has typically been studied after a very short DAPT durations,^82,83^ whereas unguided strategies have generally involved de-escalation after 1 month of event-free DAPT.^84,85^

De-escalation guided by platelet function testing was evaluated in the TROPICAL-ACS trial^82^ (2017 *n* = 2610), which enrolled patients with ACS undergoing PCI. After 1 week of DAPT with prasugrel, patients in the guided de-escalation group were switched to clopidogrel, with platelet function testing after 1 week on clopidogrel (Day 14). Guided de-escalation was non-inferior to standard DAPT for the primary NACE outcome at 12 months (CV death, MI or stroke, or BARC 2–5 bleeding). There were numerically fewer bleeding events with guided de-escalation, but this difference was not statistically significant.

A genotype-guided strategy in which carriers of CYP2C19 loss-of-function alleles received prasugrel (2%) or ticagrelor (98%), and non-carriers received clopidogrel, was evaluated in the POPular Genetics trial^83^ (2019, *n* = 2488), which enrolled patients with STEMI undergoing PCI. The genotype-guided strategy was non-inferior to standard DAPT with prasugrel or ticagrelor for the primary NACE outcome (all-cause death, MI, stroke, stent thrombosis, or major bleeding). PLATO major or minor bleeding was reduced, but no reduction in major bleeding was observed.

Unguided de-escalation from ticagrelor to clopidogrel was non-inferior and superior to standard DAPT for the primary NACE outcome (CV death, MI stroke, or BARC 2, 3, or 5 bleeding) at 12 months in the TALOS-AMI trial^84^ (2021, *n* = 2697), including patients with ACS undergoing PCI who were event-free after 1 month of ticagrelor-based DAPT. Ischaemic events were numerically lower in the de-escalation group, and bleeding, including major bleeding, was significantly reduced. A similar strategy yielded comparable results in a smaller trial, although it lacked statistical power to assess major bleeding (TOPIC 2016, *n* = 646).^86^

De-escalation of prasugrel was investigated in the HOST-REDUCE-POLYTECH-ACS trial^85^ (2020, *n* = 2338), including patients with ACS undergoing PCI. After 1 month, de-escalation from prasugrel 10 to 5 mg was non-inferior to standard DAPT with prasugrel 10 mg for the primary NACE outcome (all-cause death, MI, repeat revascularization, stroke, stent thrombosis, or BARC ≫ 2). Ischaemic events (except target-vessel revascularization) were numerically lower in the de-escalation group, and bleeding was reduced, although not major bleeding.

## Is DAPT necessary at all?

With improved stent platforms, intravascular imaging, and more potent P2Y12 inhibition, the necessity of DAPT has been questioned (*Table 11*). Pharmacological data suggest that aspirin may be redundant in the presence of the active metabolite of prasugrel.^87^

**Table 11:** Very short (a few days) or no DAPT

Trial name Year Location	Population	Age	Blinding	Strategy	Ischaemic outcome	Bleeding outcome	Met primary outcome	Comment
**ASET PILOT STUDY** **2020** **Brazil**	*n* = 201 Successful PCI with everolimus stent for CCS	60	Open-label	Single-arm Pilot study DAPT peri-PCI and loading with prasugrel at the cath lab aspirin stopped after PCI	Cardiac death, spontaneous target vessel MI, or ST at 3 months 1 Cardiac death	BARC 3 or 5 at 3 months 1 intracranial bleed	Not powered for clinical events	Stopping rule; if >3 ST occurred High-risk PCI excluded One patient with both events
**ASET-JAPAN** **2023** **Japan**	206 PCI with everolimus stent for CCS	69	Open-label	Single-arm Pilot study DAPT pre-PCI aspirin stopped after PCI prasugrel 3.75 monotherapy	Cardiac death, spontaneous target vessel MI, or ST at 3 months 0	BARC 3 or 5 at 3 months 0	Not powered for clinical events	Stopping rule based on occurrence of ST No primary events during 3 months follow-up (1 stroke and 1 non-TVR) 99.5% IVUS or OCT
**OPTICA** **2023** **Netherlands**	*n* = 75 PCI + NSTE-ACS NSTEMI 85%	65	Open-label	Single-arm Pilot study Loading with 180 mg ticagrelor or 60 mg prasugrel 2 h before PCI. Aspirin stopped after PCI Ticagrelor 85% Prasugrel 8% (DAPT 7%)	All-cause death, MI, ST or stroke at 6 months 4% (3) MI 2.7% (2) procedure-related MI at the index procedure 1.3% (1) type 2 MI	BARC 2, 3, or 5 at 6 months 9.3% (7) BARC3 or 5 2.7% (2)	Not powered for clinical events	Stopping rule; if more than one ST occurred Platelet function testing—only one patient with HPR No spontaneous MI or ST occurred
**STOP-DAPT3** **2023** **Japan**	*n* = 5966 ACS or non-ACS with HBR ACS 75% STEMI 43% or HBR 55%	72	Open-label	Monotherapy prasugrel 3.75 mg vs. DAPT with prasugrel 3.75 mg + A prasugrel loading 20 mg in both groups. Aspirin loading only in the DAPT group.	CV death, MI, stroke, or ST at 1 month 4.12% vs. 3.69%	BARC 3 or 5 at 1 month 4.47% vs. 4.71%	NO Superiority for bleeding (BARC 3 or 5) YES Non-inferiority for ischaemic outcome (CV death, MI, stroke, or ST)	Randomization before PCI 28% on aspirin->70% truly aspirin-free in the monotherapy group Low dose 3.75 mg prasugrel with loading dose 20 mg Numeric increase in MI, ST, and revascularization
**NEO-MINDSET** **2025** **Brazil**	*n* = 3410 PCI + ACS STEMI 62% NSTEMI 31%	60	Open-label	P2Y_12_ monotherapy vs. P2Y_12_ + A Prasugrel (69.2%) Ticagrelor (28.0%)	All-cause death, MI, stroke, or urgent TVR at 12 months 7.0% vs. 5.5% ST(n) 12 vs. 4	BARC 2, 3, or 5 at 12 months 2.0% vs. 4.9%	NO Non-inferiority for ischaemic outcome (All-cause death, MI, stroke, or urgent TVR) Superiority not tested for bleeding (BARC 2, 3, or 5)	Randomization within 4, median 2 days after admission, and 1 day after PCI Two hierarchical primary outcomes

A, aspirin; ACS, acute coronary syndrome; BARC, Bleeding Academic Research Consortium; C, clopidogrel; CCS, chronic coronary syndrome; CV, cardiovascular; DAPT, dual antiplatelet therapy; HBR, high bleeding risk; MI, myocardial infarction; IVUS, intravascular ultrasound; NSTEMI, non-ST-elevation myocardial infarction; OCT, optical coherence tomography; PCI, percutaneous coronary intervention; STEMI, ST-elevation myocardial infarction; ST, stent thrombosis; TVR, target vessel revascularization.

In the ASET Brazil^88^ (2020 *n* = 201) and ASET Japan^89^ (2023 *n* = 206) trials,^90^ prasugrel monotherapy at doses of 10 mg (Brazil) or 3.75 mg (Japan) was feasible and associated with low event rates in patients with CCS undergoing PCI with biodegradable-polymer platinum-chromium everolimus-eluting stents. At 3-month follow-up, only one event occurred in ASET Brazil (intracranial haemorrhage resulting in death), and no events were observed in ASET Japan. In the OPTICA trial^91^ (2023, *n* = 75), including NSTE-ACS patients undergoing PCI with DES, monotherapy with ticagrelor or prasugrel (full dose) resulted in no spontaneous MI or ST at 6 months.

In both ASET trials, and the OPTICA and TIMO trials, DAPT with aspirin and a P2Y12 inhibitor was administered during PCI, after which aspirin was discontinued. All studies were single-arm pilot trials not powered for clinical events.

Two larger trials powered for clinical events have subsequently tested strategies involving very short or no DAPT. In the NeoMindset trial^92^ (2025, *n* = 3410), patients with ACS undergoing successful PCI were randomized after a very short median DAPT duration of 2 days to monotherapy with a potent P2Y12 inhibitor (predominantly prasugrel) or standard DAPT. Monotherapy failed to meet non-inferiority for the primary outcome of all-cause death, MI, stroke, or urgent revascularization at 12 months, with all outcomes numerically higher in the monotherapy group. Major or CRNM, as well as major bleeding, at 12 months was numerically reduced by more than half; however, superiority was not formally tested because the primary outcome was not met.

A truly aspirin-free strategy without an initial period of DAPT was evaluated in the STOPDAPT3 trial^93^ (2024, *n* = 6002), which enrolled Japanese patients with ACS (75%) and/or high bleeding risk (54.5%) immediately before PCI and randomized them to either DAPT with aspirin and prasugrel or prasugrel monotherapy. A loading dose of aspirin was administered only to aspirin-naïve patients in the DAPT group, while both groups received a prasugrel loading dose of 20 mg followed by a maintenance dose of 3.75 mg. This 20/3.75 mg prasugrel regimen has been approved in Japan since 2014 based on the PRASFIT-ACS study.^94^ The co-primary bleeding endpoint at one month was not superior with prasugrel monotherapy. Although the co-primary ischaemic endpoint at 1 month met non-inferiority, ischaemic events were numerically higher in the prasugrel monotherapy group.

In conclusion, although pilot studies in low-risk patients did not show a signal of harm, two randomized trials including patients with ACS and powered for clinical events demonstrated an increase in ischaemic events, suggesting the need for an initial period of DAPT.

## Risk scores to guide DAPT duration

Given the heterogeneity in bleeding and ischaemic risk, several risk scores have been developed to support individual decision-making. The PRECISE-DAPT score^95^ (five variables) estimates 1-year bleeding risk, while the ARC-HBR criteria^96^ define high bleeding risk based on major and minor criteria (20 criteria across 12 clinical domains). Both tools can help identify patients at high bleeding risk, in whom shorter durations or less intensive antithrombotic regimens may be considered. High bleeding risk is defined as a PRECISE-DAPT score ≥25 or the presence of at least one major or two minor ARC-HBR criteria. Their discriminative capacity is modest, and evidence that the use of bleeding risk scores to guide antiplatelet therapy improves outcomes remains limited.

The DAPT score^97^ integrates both ischaemic and bleeding risk and was developed to identify patients who may benefit from or be harmed by prolonged DAPT beyond 1 year. As only one variable (age) reflects bleeding risk, its utility as bleeding risk score is limited. In the PARTHENOPE trial, use of the DAPT score improved NACE through reduction of ischaemic events.

ESC guidelines provide no graded recommendation for the use of bleeding risk scores; the PRECISE-DAPT score, DAPT score, and the ARC-HBR criteria are mentioned in the supplement. ACC/AHA guidelines mention the ARC-HBR criteria and refer to several risk tools, without naming specific scores in the text.

## Key considerations in bleeding-reduction strategies

While all bleeding-reduction strategies are successful in reducing bleeding, the main concern relates to ischaemic events. The optimal strategy should reduce bleeding without compromising ischaemic protection. Numerous trials have addressed this question, and their limitations must be carefully considered.

No single trial was powered for ischaemic events. Many trials were conducted in Southeast Asia and may not be directly generalizable to other populations, as East Asian populations have a lower risk of coronary ischaemic events but a higher risk of bleeding and stroke, the so-called East Asian paradox.^98^ In addition, some Asian trials used antiplatelet doses that are not approved outside their region.

Many trials used non-inferiority designs for MACE or NACE, with arbitrary and often wide margins. NACE may be a suboptimal endpoint in non-inferiority trials of antithrombotic therapy, as opposing directions of ischaemic and bleeding outcomes may bias results towards non-inferiority.

Low or very low ischaemic event rates were reported, particularly but not exclusively in Asian trials. This may not solely reflect the populations studied, but also the timing of randomization. For example, the TWILIGHT, ULTMATE-DAPT, and TARGET-FIRST trials randomized patients who were event-free at 1 or 3 months. Because ischaemic risk is front-loaded, such inclusion strategies result in lower event rates. However, this approach reflects clinical practice, where treatment changes at 1 to 3 months involve re-evaluation of the patient, disqualifying those who have experienced an ischaemic event, while those who have experienced a bleeding event would likely already have undergone treatment modification.

In contrast, other trials randomized patients at the index event to discontinue one of the antiplatelet agents after 1, 3, or 6 months^70,76,78,80^ following an initially identical period of DAPT in both groups. This design increases the number of events but attenuates between-group differences, which is particularly problematic in non-inferiority trials, as it may bias results towards non-inferiority.^70,78,80^ The main and landmark analyses of the SMART-CHOICE and SMART-DATE trials illustrate this effect.

All bleeding-reduction strategies for patients without an indication for OAC, except STOPDAPT-3, include an initial period of DAPT, whereas de-escalation strategies maintain DAPT while reducing the intensity of the P2Y12 inhibitor. Although pilot studies^88–90^ showed no signal of harm, the STOPDAPT-3 and NEO-MINDSET trials do not support omission of an initial period of DAPT. Accordingly, current guidelines assign a class III recommendation against de-escalation of antiplatelet therapy within the first 30 days after an ACS.^20^

## Is there a best strategy?

Although direct comparisons between the different strategies are lacking, existing evidence suggests potential differences in ischaemic risk.

### Strategies with the most consistent safety-efficacy balance

Both ticagrelor monotherapy after at least 1 month of DAPT and de-escalation strategies have consistently been shown to reduce bleeding without increasing ischaemic events.

### Strategies with ischaemic signal or uncertainty

Short DAPT followed by aspirin monotherapy reduces bleeding but increases the risk of ischaemic events, a finding also observed with longer DAPT durations in trials of prolonged DAPT. Clopidogrel monotherapy reduces bleeding but may be associated with an increased risk of ischaemic events.^81^ Prasugrel monotherapy is less well studied. STOPDAPT-3 used a very low dose of prasugrel, not approved outside Japan, and no aspirin in the monotherapy arm, except in approximately 28% of patients who were already receiving aspirin at randomization. In NEO-MINDSET,^23^ nearly 70% of patients received prasugrel, but it is unclear whether the excess ischaemic risk was driven by the choice of P2Y12 inhibitor or by the very short DAPT duration of only 2 days. Given the early ischaemic hazard also observed in other trials,^27,31,59^ this signal was likely driven by the very short duration of DAPT.

## Summary and conclusion

Following the introduction of DAPT more than two decades ago, a 12-month duration for patients with ACS was established largely by consensus rather than robust evidence and has remained the standard recommendation despite accumulating data suggesting that shorter durations are sufficient for most patients. Alternative strategies have enabled individualized treatment, but limited comparative evidence and a lack of clear guidance on strategy selection may have contributed to inertia in adopting new approaches in clinical practice. Not all bleeding-reduction strategies are equivalent. The largest body of evidence supports ticagrelor monotherapy after an initial period of DAPT, which significantly reduces bleeding without increasing ischaemic events. Timing is critical, requiring an initial period of DAPT to protect against early ischaemic events. Considering these findings, updates are anticipated in the forthcoming 2027 ESC ACS guidelines.

## Lead author biography


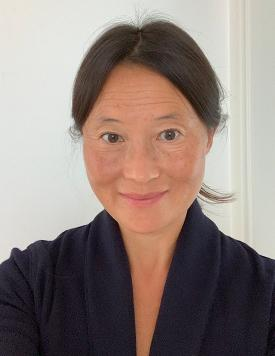


Moa Simonsson is a consultant cardiologist at the Cardiac ICU at Karolinska University hospital and a post-doctoral researcher at Karolinska Institutet, Stockholm, Sweden. She is the chair of the Swedish Society of Cardiology working group of Acute Cardiac care and Ischemia, and the National representative for Sweden in the ESC working group for Acute CardioVascular Care.

## Data Availability

No new data were generated or analysed in support of this research.
